# Itraconazole inhibits endothelial cell migration by disrupting inositol pyrophosphate-dependent focal adhesion dynamics and cytoskeletal remodeling

**DOI:** 10.1016/j.biopha.2023.114449

**Published:** 2023-05

**Authors:** Ji Qi, Weiwei Cheng, Zhe Gao, Yuanyuan Chen, Megan L. Shipton, David Furkert, Alfred C. Chin, Andrew M. Riley, Dorothea Fiedler, Barry V.L. Potter, Chenglai Fu

**Affiliations:** a The province and ministry co-sponsored collaborative innovation center for medical epigenetics, Tianjin Key Laboratory of Metabolic Diseases, Department of Physiology and Pathophysiology, Tianjin Medical University, Tianjin 300070, China; b Institute for Developmental and Regenerative Cardiovascular Medicine, Xinhua Hospital, Shanghai Jiao Tong University School of Medicine, Shanghai 200092, China; c School of Integrative Medicine, Tianjin University of Traditional Chinese Medicine, Tianjin 301617, China; d Medicinal Chemistry & Drug Discovery, Department of Pharmacology, University of Oxford, Mansfield Road, Oxford OX1 3QT, UK; e Leibniz-Forschungsinstitut für Molekulare Pharmakologie, Berlin, Germany; f Weill Cornell/Rockefeller/Sloan Kettering Tri-Institutional MD-PhD Program, New York, NY, USA

**Keywords:** Itraconazole, IP6K, Inositol pyrophosphate, FAK, α-actinin, Arp2/3

## Abstract

The antifungal drug itraconazole has been repurposed to anti-angiogenic agent, but the mechanisms of action have been elusive. Here we report that itraconazole disrupts focal adhesion dynamics and cytoskeletal remodeling, which requires 5-diphosphoinositol 1,2,3,4,6-pentakisphosphate (5-InsP_7_). We find that inositol hexakisphosphate kinase 1 (IP6K1) binds Arp2 and generates 5-InsP_7_ to recruit coronin, a negative regulator of the Arp2/3 complex. IP6K1 also produces focal adhesion-enriched 5-InsP_7_, which binds focal adhesion kinase (FAK) at the FERM domain to promote its dimerization and phosphorylation. Itraconazole treatment elicits displacement of IP6K1/5-InsP_7_, thus augments 5-InsP_7_-mediated inhibition of Arp2/3 complex and reduces 5-InsP_7_-mediated FAK dimerization. Itraconazole-treated cells display reduced focal adhesion dynamics and actin cytoskeleton remodeling. Accordingly, itraconazole severely disrupts cell motility, an essential component of angiogenesis. These results demonstrate critical roles of IP6K1-generated 5-InsP_7_ in regulating focal adhesion dynamics and actin cytoskeleton remodeling and reveal functional mechanisms by which itraconazole inhibits cell motility.

## Highlights

• IP6K1 binds Arp2 and generates 5-InsP_7_ to recruit coronin to Arp2/3 complex.
• IP6K1 generates 5-InsP_7_ in focal adhesion to promote FAK dimerization.
• Itraconazole inhibits Arp2/3 complex by enhancing the binding of IP6K1 to Arp2.
• Itraconazole lowers FAK phosphorylation by removing IP6K1 from focal adhesion.
• Itraconazole disrupts 5-InsP_7_-mediated focal adhesion and cytoskeletal dynamics.

## 1 Introduction

Itraconazole is a widely used antifungal drug with a clinical history of over 30 years. Recently, itraconazole is being repurposed as an anti-angiogenic agent and exhibits therapeutic efficacies in cancer [1], hereditary hemorrhagic telangiectasia [2], and ocular neovascularization [3]. Despite the identification of many itraconazole target proteins [4], [5], [6], the downstream functional mechanisms by which itraconazole inhibits angiogenesis are unknown.

Angiogenesis is a complex morphogenetic process, requiring endothelial cell proliferation and migration to form vascular structures. The actin-related protein 2/3 (Arp2/3) complex generates dendritic actin networks at the cell cortex that produce the driving force for lamellipodia protrusion [7]. In mammalian cells, the Arp2/3 complex consists of two actin-related proteins, Arp2 and Arp3, and five subunits [7]. Coronin binds the p34 subunit of the Arp2/3 complex and inhibits its ability to nucleate new actin filaments [8], [9].

Focal adhesions are plaque-like structures that link the actin cytoskeleton to extracellular matrix. Focal adhesions are composed of multiple layers of proteins such as *α*-actinin, focal adhesion kinase (FAK) and vinculin [10]. *α*-Actinin is crucial for formation of actin bundles and links actin to focal adhesions, which contributes to focal adhesion maturation [11]. FAK is activated by phosphorylation and plays essential roles in focal adhesion turnover, which is critical for cell migration and blood vessel formation [12], [13].

5-InsP_7_ is a signalling molecule generated by IP6Ks and mediates diverse cellular processes, such as mRNA stability [14], protein secretion [15] and cellular energy homeostasis [16]. 5-InsP_7_ regulates target proteins by binding or pyrophosphorylation [17]. Because 5-InsP_7_ is metabolized extremely rapidly and previous studies suggesting discrete intracellular 5-InsP_7_ pools [18], [19], [20], synthesis of 5-InsP_7_ likely needs to occur proximal to its sites of actions.

In this study, we demonstrate that IP6K1 generates a local pool of 5-InsP_7_ to regulate FAK dimerization and the Arp2/3 complex. Itraconazole treatment dislocates IP6K1 and thus disrupts 5-InsP_7_-mediated focal adhesion dynamics and actin cytoskeleton remodelling.

## 2 Materials and methods

### 2.1 Materials

5-PCF_2_Am-InsP_5_ (CF2) was synthesized as previously described [21]. 5-InsP_7_ and 5-PCP-InsP_5_ (5-PCP) synthesized using similar methods to those previously described [22], [23], [24], [25]. All synthetic compounds were purified by ion-exchange and/or RP-18 chromatography and were fully characterized by ^1^H, ^31^ P, and ^13^ C nuclear magnetic resonance spectroscopy.

Anti-IP6K1, anti-myc, anti-coronin 1B, anti-Arp3, anti-cadherin antibodies were from Santa Cruz Biotechnology. Anti-Arp2, anti-p34 antibodies were from Bethyl Laboratories. Anti-flag, anti-vinculin antibodies were from Sigma-Aldrich. Anti-α-actinin, anti-FAK, anti-paxillin, anti-phospho-paxillin, anti-β-actin antibodies were from Cell Signaling Technology. Anti-phospho-FAK (Y397) antibody was from Abcam. Anti-GST antibody was from Proteintech.

HEK293, HEK293T/17 cell lines were from ATCC, HUVECs were from Lonza.

### 2.2 Cell culture

Human embryonic kidney (HEK) 293 cells, HEK 293 T/17 cells, wild type (WT) mouse embryonic fibroblast (MEF) cells and *IP6K1* knockout (KO) MEF cells were cultured in DMEM medium (Biosharp Life Sciences) supplemented with 10% (v/v) FBS (Thermo Fisher Scientific), 100 U/ml penicillin and 100 ug/ml streptomycin (Yeasen). Human umbilical vein endothelial cells (HUVECs) were cultured in EGM2 medium (Lonza). All cells were maintained at 37 °C with 5% CO_2_. Cells were plated one day before experiments. Before treating cells, the existing cell culture medium was exchanged with fresh medium. Transfections were conducted with Lipofectamine 3000 (Thermo Fisher Scientific).

### 2.3 Western blotting

Cells were lysed in lysis buffer containing 50 mmol/L Tris-HCl (pH 7.4), 100 mmol/L NaCl, 0.5% Igepal CA630, 5 mmol/L MgCl_2_ and protease/phosphatase inhibitors (Yeasen). Lysates were pulse sonicated and centrifuged at 14,000 g for 10 min at 4ºC. Protein concentrations were normalized using a Pierce BCA Protein Assay Kit (Thermo Fisher Scientific). SDS loading buffer (Thermo Fisher Scientific) containing 5% β-mercaptoethanol was added, and the samples were boiled for 5 min. Proteins were separated by 8–15% SDS-PAGE gel and transferred to a PVDF membrane (Thermo Fisher Scientific). The membrane was blocked with 5% non-fat dry milk in Tris-buffered saline containing 0.1% Tween 20 (TBST) at room temperature for 1 h, and was incubated with primary antibody overnight at 4 °C. The membrane was washed three times with TBST and incubated with HRP-conjugated secondary antibody for 1 h at room temperature followed by three washes with TBST. Immobilon Western Chemiluminescent HRP substrate (Thermo Fisher Scientific) was used to detect the signal of the secondary antibody. The membrane was then imaged using ChemiDoc™ imaging system (Bio-Rad).

### 2.4 Immunoprecipitation

Cells were lysed in the lysis buffer containing 50 mmol/L Tris-HCl (pH 7.4), 100 mmol/L NaCl, 0.5% Igepal CA630, 5 mmol/L MgCl_2_ and protease/phosphatase inhibitors (Yeasen). Lysates were passed through 30gauge needles 20times and centrifuged at 14,000 g for 10 min at 4ºC. The supernatants were collected and pre-cleaned with protein A/G beads (Santa Cruz Biotechnology) for 90 min at 4ºC. Lysates were centrifuged briefly, and the supernatants were collected while the protein A/G beads were discarded. Primary antibody was added to cell lysates and incubated at 4ºC overnight. Protein A/G beads were then added to the cell lysates and incubated for 2 h at 4ºC. The beads were washed with cold lysis buffer 3 times. 1.5x SDS loading buffer containing 5% β-mercaptoethanol was added, and the samples were boiled for 5 min.

### 2.5 Subcellular fractionation

Cell membrane fractions were isolated by ultracentrifuge. Cells were homogenized at 4ºC in buffer containing 250 mM Sucrose, 20 mM HEPES, PH 7.4, 10 mM KCl, 1.5 mM MgCl_2_, 1 mM EDTA, 1 mM EGTA, 1 mM DTT and protease/phosphatase inhibitors. Lysates were passed through a 30-gauge needle 20 times, incubated on ice for 20 min, and centrifuged at 14,000 g for 20 min at 4ºC. The supernatant was collected and centrifuged at 200,000 g for 2 h at 4ºC. The resulting supernatant was the cytosolic fraction. The pellet left was washed with fractionation buffer, re-suspended by pipetting, and re-centrifuged at 200,000 g for 2 h at 4ºC. The resulting pellet was the membrane fraction, which contains plasma membrane, microsomes, and small vesicles.

### 2.6 Immunostaining

Cultured cells were washed with PBS followed by fixation with 4% (w/v) paraformaldehyde. The samples were blocked with 10% (v/v) goat serum (MilliporeSigma) for 10 min at room temperature then incubated with primary antibodies at 4 °C overnight. The samples were washed multiple times with PBS for 3 h at room temperature then incubated with fluorescent-dye conjugated secondary antibodies for 1 h at room temperature. Nuclei were stained with Hoechst 33342 (Thermo Fisher Scientific) for 10 min. F-actin was stained by incubating with fluorescein labeled phalloidin (Thermo Fisher Scientific) for 30 min. Slices were mounted with ProLong Gold Antifade Mountant (Thermo Fisher Scientific). Pictures were taken under a confocal microscope (Zeiss LSM 800).

### 2.7 Plasmid cloning

Flag-tagged Arp2, myc-tagged WT IP6K1, flag-tagged p34, GST-fused coronin, GST-fused IP6K1, flag-tagged α-actinin, flag-tagged FAK, GST-fused FAK, GST-fused FAK FERM domain, myc-tagged kinase defective mutant (mut) IP6K1 were cloned into the pCDH-EF1α-MCS-T2A-GFP vector (System Biosciences). The PCR products were generated by using Phusion Polymerase (Thermo Fisher Scientific) and inserted into vectors using In-Fusion HD Enzyme (Takara Bio). All newly constructed plasmids were sequence-verified.

### 2.8 Lentivirus generation

HEK 293 T/17 cells were plated one day before experiments and allowed to grow to 70% confluence. Lentiviral vectors harboring the gene of interest together with pMD2. G and psPAX2 were transfected into HEK 293 T/17 cells using Lipofectamine 3000. Cell culture medium was replaced with fresh medium 4 h after transfection. The virus containing medium was collected 48 h later and filtered through a 0.45 µm filter then mixed with 1/2 vol of concentration medium containing 25.5% PEG 6000 (MilliporeSigma), 0.9 M NaCl, 2.5 mM Na_2_HPO_4_, and 0.4 mM KH_2_PO_4_. The samples were stored at 4ºC overnight then centrifuged at 17,000 g for 1 h at 4ºC. The resulting pellet containing lentivirus was resuspended with DMEM medium and stored at − 80ºC.

### 2.9 *In vitro* enzymatic assays

IP6K1 activity was measured using an ADP-Glo™ Max Assay Kit (Promega). GST-fused IP6K1 was produced in HEK 293 cells and harvested by Glutathione Sepharose. PreScission Protease was used to cleave the GST and release IP6K1 into a buffer containing 50 mM Tris (pH 7.4), 10 mM MgCl_2_, and 2.5 mM DTT. IP6K1 activity assay was conducted in a reaction containing ∼20 mg/ml protein, 50 μM InsP_6_, and 100 μM ATP for 2 h in 37ºC. Itraconazole or DMSO was added to the reaction. The reaction was quenched with ADP-Glo Reagent for 40 min, and ADP-Glo Max Detection Reagent was added and incubated for 60 min. Using an opaque white 96-well plate (Costar), the bioluminescent signal in relative light units was obtained on a microplate reader with an integration time of 1 s per well.

### 2.10 *In vitro* binding assay

To assess the binding of p34 with coronin, flag-tagged p34 and GST-fused coronin were produced in HEK293 cells. Cell lysates were pre-cleaned with protein A/G beads (Santa Cruz), anti-flag-tag antibody was added to cell lysates expressing flag-p34 overnight. Flag-p34 was pulled down by protein A/G beads and washed for three times. Separately, Glutathione Sepharose (GE Life Sciences) was used to pull down GST-fused coronin. GST-coronin was released by reduced glutathione (50 mM) in a buffer containing 200 mM NaCl, 50 mM Tris (PH 9.0) and protease inhibitors. Purified coronin was added to protein A/G agarose bound flag-tagged p34 in the presence of InsP_6_ or 5-InsP_7_ or 5-PCP or CF2 overnight at 4 °C. Beads were then collected and washed three times. The samples were loaded with 1.5x SDS loading buffer containing 5% β-mercaptoethanol and boiled for 5 min.

To assess the binding of flag-FAK with GST-FAK, flag-FAK and GST-FAK were produced in HEK293 cells. Cell lysates were pre-cleaned with protein A/G beads (Santa Cruz), anti-flag antibody was added to cell lysates expressing flag-FAK overnight. Flag-FAK was pulled down by protein A/G beads and washed three times. Separately, Glutathione Sepharose (GE Life Sciences) was used to pull down GST-FAK. GST-FAK was released by reduced glutathione (50 mM) in a buffer containing 200 mM NaCl, 50 mM Tris (PH 9.0) and protease inhibitors. Purified GST-FAK was added to protein A/G agarose bound flag-FAK in the presence of InsP_6_ or 5-InsP_7_ or 5-PCP or CF2 overnight at 4 °C. Beads were then collected and washed three times. The samples were loaded with 1.5x SDS loading buffer containing 5% β-mercaptoethanol and boiled for 5 min.

To assess binding of p34, coronin, FAK and FAK FERM domain to 5-PCP-InsP_5_, the control and 5-PCP-InsP_5_ resin were equilibrated with cell lysis buffer. Purified flag-p34, GST-coronin, GST-FAK and GST-FAK FERM domain were added to the 5-PCP-InsP_5_ resin and incubated overnight at 4 °C. The resins were collected and washed three times. The samples were mixed with 1.5x SDS loading buffer containing 5% β-mercaptoethanol and boiled for 5 min.

Flag-p34 was over-expressed in HEK 293 cells and pulled down by anti-flag antibody and protein A/G beads. Flag-p34 was then released by 3x DYKDDDDK peptide (Thermo Fisher Scientific) in a buffer containing 50 mmol/L Tris-HCl (pH 7.4), 100 mmol/L NaCl, 0.5% Igepal CA630, 5 mmol/L MgCl_2_ and protease/phosphatase inhibitors.

GST-coronin, GST-FAK and GST-FAK FERM domain were produced in HEK 293 cells and pulled down by Glutathione Sepharose. The GST-fused proteins were then released by reduced glutathione (50 mM) in a buffer containing 200 mM NaCl, 50 mM Tris (PH 9.0) and protease inhibitors.

### 2.11 Live cell imaging

LifeAct-mScarlet or GFP expressing HUVECs or MEF cells were plated onto a glass bottom cell culture dish. Cell images were taken by utilizing a confocal microscope (ZEISS 800) that took one picture per minute for 30 min.

### 2.12 Quantification and statistical analysis

Experiments were repeated for five times. Image J was used to quantify western blots, band intensities were normalized to total or control protein. Volocity software (V6.3, PerkinElmer Inc.) was used to quantify the immunofluorescence data. Statistical analysis was done with Graphpad Prism 7. Data represent means ± standard error of mean (SEM), n = number of independent repeats. Difference between two groups was analyzed by unpaired two-tailed Student's *t*-test. The Pearson correlation coefficient was used to quantify the degree of colocalization between two proteins. Significance is defined as ∗p < 0.05; ∗∗p < 0.01; ∗∗∗p < 0.001.

## 3 Results

### 3.1 Itraconazole enhances binding of IP6K1 to Arp2

Endothelial cell migration, an essential component for angiogenesis is severely inhibited by itraconazole (Fig S1A, B). Arp2/3 complex-mediated lamellipodia formation is required for cell migration[26]. Immunoprecipitations and western blots reveal that itraconazole does not affect the binding between Arp2 and Arp3, two major components of Arp2/3 complex (Fig S2A, B).

**Fig. 1:**
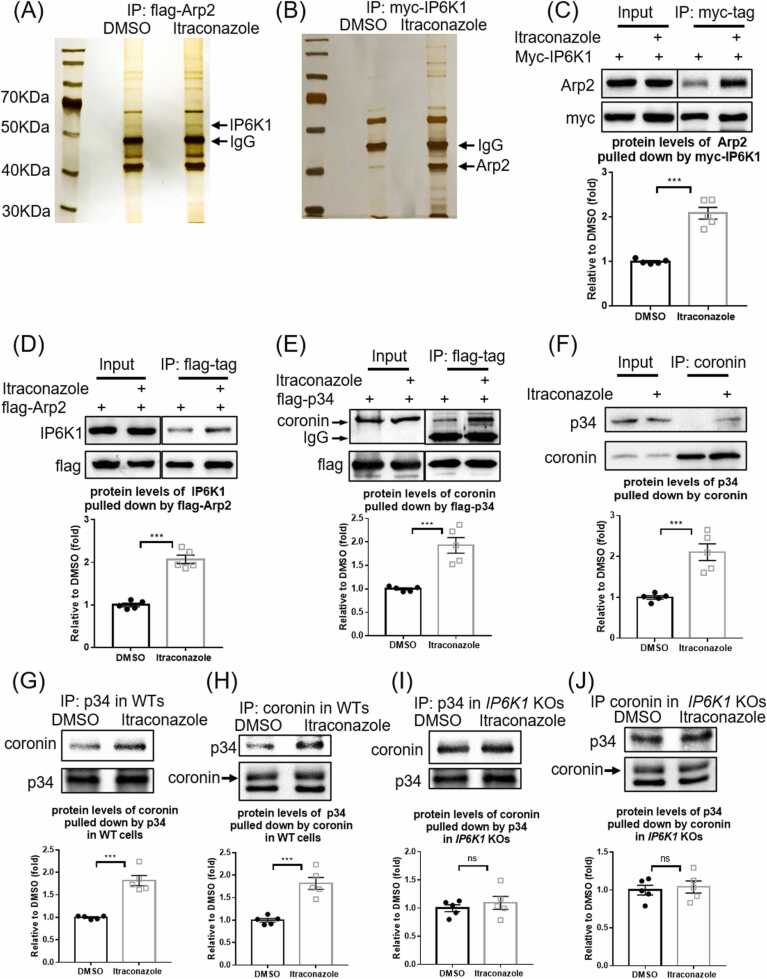
Itraconazole strengthens interactions of Arp2/3 complex with IP6K1 and coronin. (A) Flag-tagged Arp2 (flag-Arp2) was over-expressed, and the cells were treated with itraconazole (3 μM) for 24 h. Immunoprecipitation of flag-Arp2 and silver staining reveal that a protein at ∼55 kDa, which was identified as IP6K1, is increased in the itraconazole preparations (arrow). (B) Myc-tagged IP6K1 (myc-IP6K1) was over-expressed, and the cells were treated with itraconazole (3 μM) for 24 h. Immunoprecipitation of myc-IP6K1 and silver staining show that a protein at ∼40 kDa, which was identified as Arp2, is increased in the itraconazole preparations (arrow). (C) Pulling down myc-IP6K1 co-precipitates more Arp2 in the itraconazole treated cells. (D) Immunoprecipitation of flag-Arp2 co-pulls down more IP6K1 in the itraconazole preparations. (E) Cells expressing flag-tagged p34 (flag-p34) were treated with itraconazole (3 μM) for 24 h. Immunoprecipitation of flag-p34 co-pulled down more coronin in itraconazole treated cells. (F) Immunoprecipitation of endogenous coronin co-pulls down more p34 in the itraconazole treated HUVECs. (G) Immunoprecipitation of p34 co-pulls down more coronin in itraconazole treated WT MEF cells. (H) Pulling down coronin co-pulls down more p34 in itraconazole treated WT MEF cells. (I) Immunoprecipitation of p34 co-pulls down similar amounts of coronin in DMSO and itraconazole treated *IP6K1* KO MEF cells. (J) Pulling down coronin co-pulls down similar amounts of p34 in DMSO and itraconazole treated *IP6K1* KO MEF cells. Statistical data are presented as mean ± SEM, Student’s *t*-test, n = 5 independent repeats, * **p < 0.001, ns=not significant.

We examined whether itraconazole alters proteins interactions with Arp2. Immunoprecipitation of flag-Arp2 co-pulls down several proteins revealed by silver staining (Fig. 1A). A protein band with molecular weight ∼55KDa, which was identified as IP6K1 by mass spectrometry, is enriched in the itraconazole-treated samples, suggesting that its binding to Arp2 is enhanced (Fig. 1A). The interaction of IP6K1 with Arp2 is validated (Fig. 1B), and the itraconazole-induced enhanced binding of IP6K1 with Arp2 is confirmed by western blot (Fig. 1C, D).

### 3.2 Itraconazole enhances 5-InsP_7_-mediated recruitment of coronin to the Arp2/3 complex

Itraconazole treatment does not affect IP6K1 protein levels nor the enzymatic activity of IP6K1 (Fig S3A-C). Previous studies suggest a local pool model whereby 5-InsP_7_ functions in discrete, localized subcellular areas where specific IP6Ks are enriched [18], [19], [20]. We hypothesize that itraconazole-induced recruitment of IP6K1 to Arp2 may catalyze 5-InsP_7_-mediated regulation of the Arp2/3 complex, and ask whether 5-InsP_7_ regulates the interaction of the p34 subunit of Arp2/3 complex with coronin, an inhibitor of the Arp2/3 complex [9], [27], because this interaction is strengthened by itraconazole (Fig. 1E, F).

To test whether IP6K1/5-InsP_7_ play a role in mediating the binding of p34 with coronin, we performed immunoprecipitations of endogenous p34 and coronin in WT and *IP6K1* KO MEF (mouse embryonic fibroblast) cells (Fig. 1G-J). The results show that the binding between p34 and coronin is increased by itraconazole treatment in WT but not *IP6K1* KO preparations (Fig. 1G-J), indicating that IP6K1 is involved in itraconazole-induced augmentation of p34/coronin interaction.

**Fig. 2:**
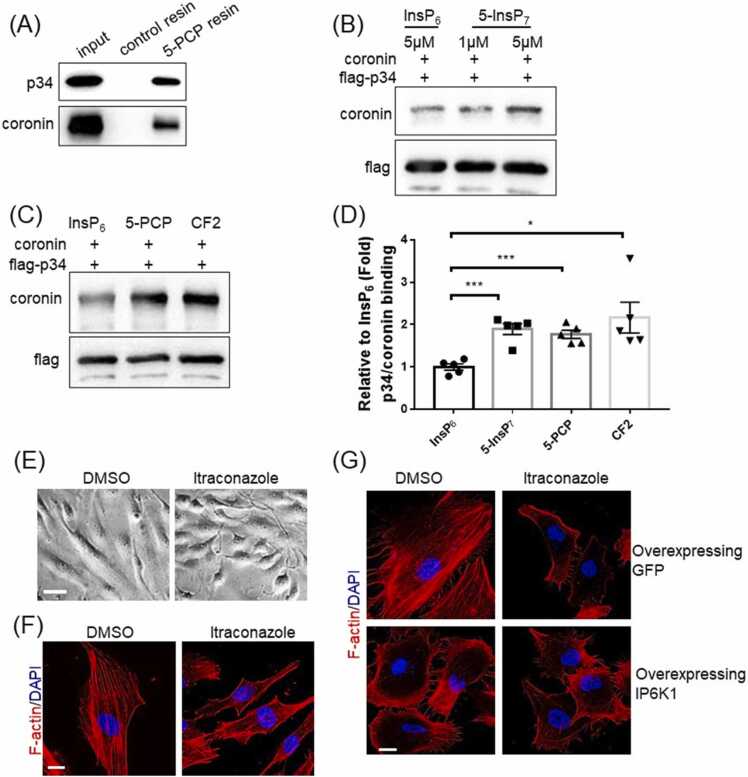
Itraconazole enhances 5-InsP_7_-mediated recruitment of coronin to Arp2/3 complex. (A) 5-PCP-InsP_5_ (5-PCP) resin pulls down endogenous coronin and p34 in the whole cell lysates of HUVECs. (B, C) Flag-p34 immobilized on protein A/G beads was incubated with coronin in an *in vitro* protein binding assay. (B) Compared with InsP_6_, 5-InsP_7_ enhances the binding between p34 and coronin. (C) Compared with InsP_6_, both 5-PCP and CF2 enhance the binding between p34 and coronin. (D) Statistical analysis of binding of coronin with p34. Statistical data are presented as mean ± SEM, Student’s *t*-test, n = 5 independent repeats, *p < 0.05, * **p < 0.001. (E, F) HUVECs were treated with DMSO or itraconazole (3 μM) for 24 h. (E) Itraconazole treated cells display smaller and irregular shapes. Scale bar 50 µm. (F) Phalloidin staining shows that F-actin is widely distributed in the control cells, but is largely accumulated at the cell cortex in the itraconazole treated cells. Scale bar 20 µm. (G) F-actin is mainly at the cell cortex of IP6K1-overexpressing cells, which is similar to itraconazole treatment. Scale bar 20 µm.

We examined the role of 5-InsP_7_, the major product of IP6K1 by testing whether 5-InsP_7_ physically binds p34 and/or coronin. We utilized an affinity resin containing immobilized 5-PCP-InsP_5_ (5-PCP) [28], a nonhydrolyzable bisphosphonate analog of 5-InsP_7_ as a bait to pull down p34 and coronin (Fig. 2A, Fig S4A). 5-PCP resin pulls down endogenous p34 and coronin in the whole cell lysates of HUVECs (Fig. 2A), and also pulls down purified p34 and coronin in an *in vitro* protein binding assay (Fig S4A), suggesting that 5-InsP_7_ directly binds p34 and coronin.

We have previously demonstrated that 5-InsP_7_ binding can facilitate protein-protein interactions [20], and ask whether 5-InsP_7_ mediates the interaction between p34 and coronin. The *in vitro* protein binding assays show that 5-InsP_7_ enhances the binding of p34 with coronin (Fig. 2B, D). Both 5-PCP (5-PCP-InsP_5_) and CF2 (5-PCF_2_Am-InsP_5_), nonhydrolyzable analogs of 5-InsP_7_ and structurally closely mimic the physicochemical and biochemical properties of 5-InsP_7_ [21], [29], enhance the interaction between p34 and coronin (Fig. 2 C, D). Itraconazole treatment does not seem to affect the expression levels of Arp2, Arp3, or p34 (Fig S4B). The expression levels of coronin are 30% lower in the itraconazole treated cells (Fig S4B).

Consistent with Arp2/3 complex inhibition, itraconazole treatment elicits prominent morphological changes in HUVECs. The DMSO-treated control cells are cobblestone-like, whereas the itraconazole-treated cells display smaller and more irregular shapes (Fig. 2E). Fluorescein phalloidin staining reveals drastically different actin filament architectures in DMSO- and itraconazole-treated HUVECs (Fig. 2F). While F-actin is widely distributed in the DMSO-treated control cells, it largely accumulates at the cell cortex in the itraconazole-treated cells (Fig. 2F), which is similar to Arp2/3 complex inhibition (Fig S5) [30]. Increasing 5-InsP_7_ levels by overexpressing IP6K1 in HUVECs, which presumably strengthens the interactions of coronin with Arp2/3 complex, elicits similar effects on F-actin distribution as itraconazole does (Fig. 2G). We utilized WT and *IP6K1* KO MEF cells to validate that IP6K1 is involved in itraconazole-elicited alteration of F-actin (Fig S6). Deletion of IP6K1 impairs F-actin formation [31]. Itraconazole disrupts F-actin in WT cells but does not further disrupt F-actin in *IP6K1* KO cells (Fig S6).

### 3.3 Itraconazole dissociates IP6K1/α-actinin from focal adhesions

**Fig. 3:**
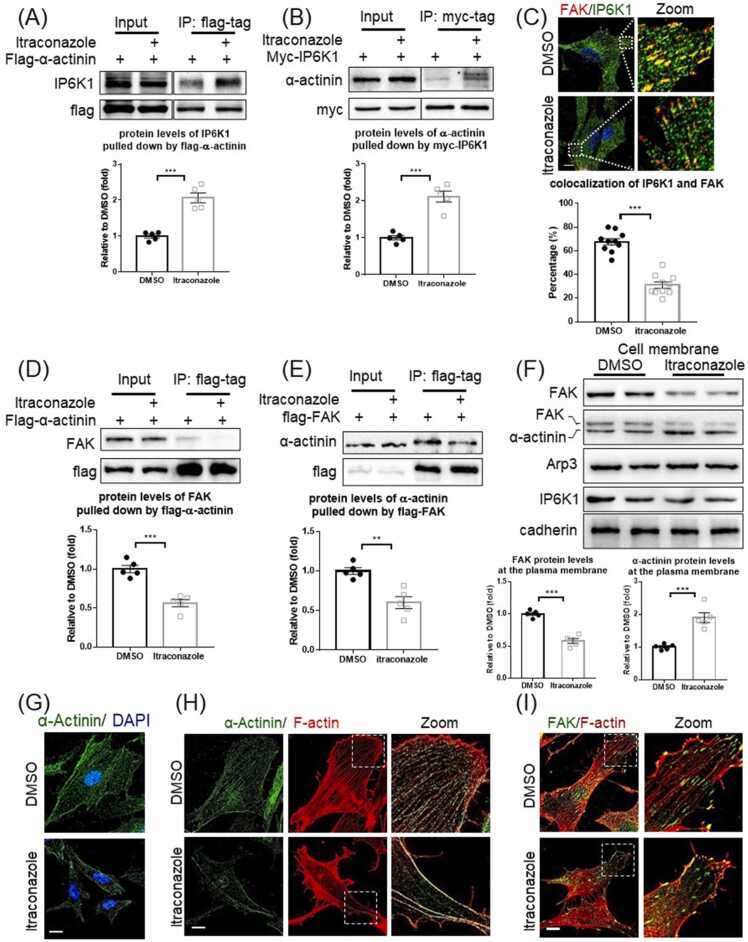
Itraconazole dismisses IP6K1/α-actinin from focal adhesions. (A) Flag-α-actinin was overexpressed, and the cells were treated with DMSO or itraconazole (3 μM) for 24 h. Immunoprecipitation of flag-α-actinin co-pulls down more IP6K1 in itraconazole-treated cells. (B) Myc-IP6K1 was overexpressed, and the cells were treated with DMSO or itraconazole (3 μM) for 24 h. Pulling down myc-IP6K1 co-precipitates more α-actinin in itraconazole treated cells. (C) Confocal microscopy shows that itraconazole treatment disrupts the colocalization of IP6K1 and FAK. Scale bar 20 µm. 10 images from 5 independent experiments were analyzed. (D) Flag-α-actinin was overexpressed, and the cells were treated with DMSO or itraconazole (3 μM) for 24 h. Immunoprecipitation of flag-α-actinin co-pulls down less FAK in itraconazole-treated cells. (E) Flag-FAK was overexpressed, and the cells were treated with DMSO or itraconazole (3 μM) for 24 h. Pulling down flag-FAK co-precipitates less α-actinin in itraconazole-treated cells. (F) HUVECs were treated with DMSO or itraconazole (3 μM) for 24 h. The cell membrane fractions were isolated. Itraconazole treatment increases α-actinin but decreases FAK protein levels in the cell membrane fractions. (G) Confocal microscopy reveals that α-actinin is widely distributed in DMSO treated control cells, but is localized mostly at the cell border in the itraconazole treated cells. Scale bar 20 µm. (H) Confocal microscopy shows that both α-actinin and F-actin are reduced in the cytosol of itraconazole treated cells. Scale bar 20 µm. (I) Confocal microscopy shows that a large portion of FAK is not connected with F-actin in the itraconazole treated cells. Scale bar 20 µm. Statistical data are presented as mean ± SEM, Student’s *t*-test, n = 5 independent repeats, * *p < 0.01, * **p < 0.001.

We previously reported that IP6K1 binds α-actinin, which plays a critical role in regulating focal adhesion turnover [31], and ask whether itraconazole affects the interaction of IP6K1 with α-actinin. Immunoprecipitations reveal that the interaction of IP6K1 with α-actinin is strengthened by itraconazole treatment (Fig. 3A, B).

We ask whether the increased binding of IP6K1 to α-actinin induced by itraconazole intensifies IP6K1 at focal adhesions. Unexpectedly, confocal microscopy shows that the co-localization of IP6K1 with FAK is decreased after itraconazole treatment (Fig. 3C). These results suggest that IP6K1 is removed by itraconazole from focal adhesions. This prompted us to assess the interaction between α-actinin and FAK because IP6K1 indirectly associates with FAK through α-actinin [32]. Immunoprecipitation studies reveal that the binding between α-actinin and FAK is disrupted by itraconazole treatment (Fig. 3D, E).

Itraconazole treatment does not seem to affect the expression levels of α-actinin and FAK (Fig S7A). However, itraconazole causes enrichment of α-actinin in the cell membrane, but decreases FAK protein levels in the cell membrane (Fig. 3F). This result further suggests that the interaction of α-actinin with FAK is disrupted by itraconazole. IP6K1 does not seem to play a role in the itraconazole-induced redistribution of α-actinin. Neither overexpression nor knocking down of IP6K1 increases the protein levels of α-actinin in the plasma membrane fractions (Fig S7B, C).

We utilized confocal microscopy to confirm that itraconazole elicits redistribution of α-actinin (Fig. 3G). In control cells, α-actinin appears diffuse and evenly distributed. In itraconazole-treated cells, α-actinin distributes more heavily along the cell border (Fig. 3G). Consistent with the redistribution of α-actinin, double staining of α-actinin and F-actin reveals that the stress fibers formation is drastically reduced in itraconazole-treated cells (Fig. 3H). Coherently, a large portion of focal adhesions are assembled without connection with stress fibers in itraconazole treated cells (Fig. 3I).

### 3.4 Itraconazole disrupts 5-InsP_7_-mediated FAK phosphorylation and dimerization

**Fig. 4:**
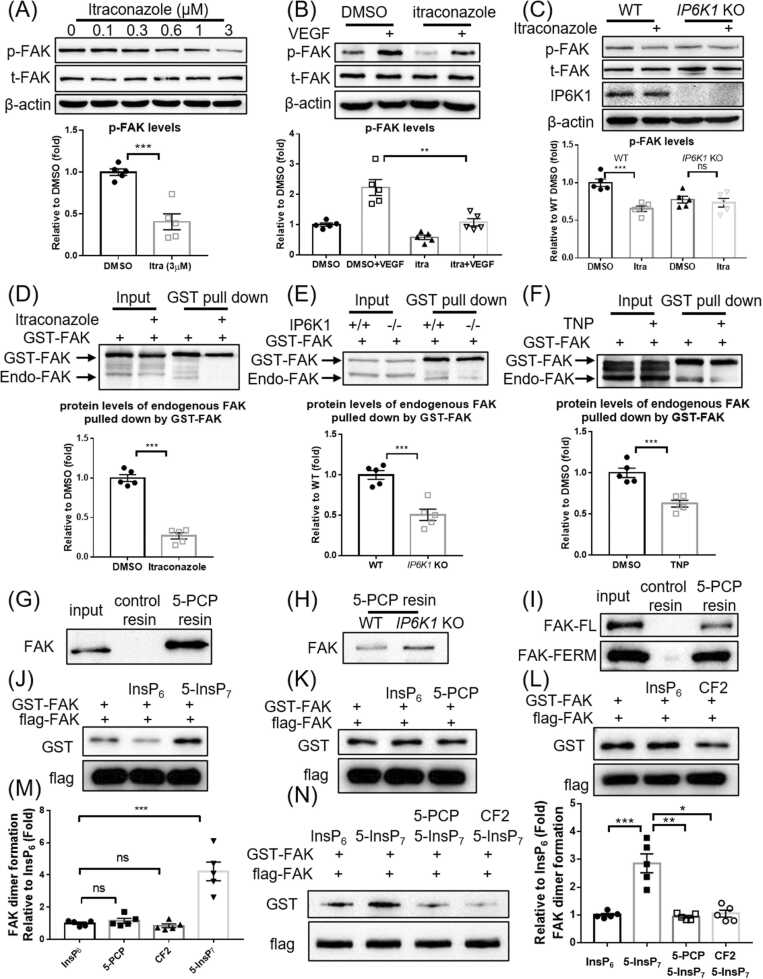
Itraconazole disrupts 5-InsP_7_-mediated FAK dimerization and autophosphorylation. (A) HUVECs were treated with itraconazole for 24 h. Itraconazole inhibits FAK phosphorylation (Y397). (B) HUVECs were treated with itraconazole (3 μM) for 24 h. The cells were then treated with VEGF (20 ng/ml). Itraconazole inhibits VEGF-induced FAK phosphorylation (Y397). (C) WT and *IP6K1* KO MEF cells were treated with itraconazole (3 μM) for 24 h. FAK phosphorylation (Y397) levels are lower in the *IP6K1* KOs. Itraconazole decreases FAK phosphorylation (Y397) levels in the WTs but not *IP6K1* KOs. (D) HUVECs expressing GST-FAK were treated with DMSO or itraconazole (3 μM) for 24 h. GST-FAK co-precipitates substantially less endogenous FAK in the itraconazole-treated cells. (E) GST-FAK was overexpressed in WT and *IP6K1* KO MEF cells. GST-FAK co-precipitates less endogenous FAK in *IP6K1* KOs than that of WTs. (F) HUVECs expressing GST-FAK were treated with DMSO or TNP (5 μM) for 24 h. GST-FAK co-pulls down less endogenous FAK in the TNP-treated cells. (G) 5-PCP resin pulls down endogenous FAK in HUVECs. (H) 5-PCP resin pulls down endogenous FAK in WT and *IP6K1* KO MEF cells. (I) 5-PCP resin pulls down purified full length (FL) FAK and FAK FERM domain. (J-L) Flag-FAK immobilized on protein A/G beads was incubated with GST-FAK in an *in vitro* protein binding assay. (J) Compared with InsP_6_, 5-InsP_7_ enhances the binding of flag-FAK to GST-FAK. (K) 5-PCP does not enhance the binding of flag-FAK to GST-FAK. (L) CF2 does not increase the binding of flag-FAK to GST-FAK. (M) Statistical analysis of the binding between flag-FAK and GST-FAK in (J-L). (N) Flag-FAK immobilized on protein A/G beads was incubated with GST-FAK in the presence of InsP_6_ (5 μM) or 5-InsP_7_ (5 μM) or 5-PCP (10 μM) + 5-InsP_7_ (5 μM) or CF2 (10 μM) + 5-InsP_7_ (5 μM). 5-PCP and CF2 block 5-InsP_7_-mediated FAK dimerization. Statistical data are presented as mean±SEM, Student’s *t*-test, n = 5 independent repeats, ns=not significant, * p < 0.05, * *p < 0.01, * **p < 0.001.

5-InsP_7_ generated by IP6K1 in focal adhesions is important for FAK phosphorylation [31], [33], which is confirmed in this study (Fig S8A-C). Relocating IP6K1 from focal adhesions by itraconazole results in reduced FAK phosphorylation (Fig. 4A). Itraconazole also blocks FAK phosphorylation induced by VEGF (Fig. 4B).

We utilized WT and *IP6K1* KO MEF cells to confirm that depleting IP6K1-generated 5-InsP_7_ in focal adhesions is responsible for itraconazole-induced inhibition of FAK phosphorylation (Fig. 4C). Western blots show that FAK phosphorylation levels are lower in *IP6K1* KO cells than that of WT cells. Itraconazole decreases FAK phosphorylation in WT cells to a level similar to that in the *IP6K1* KO cells. In *IP6K1* KO cells, itraconazole barely reduces FAK phosphorylation levels (Fig. 4C).

FAK dimerization is critical for its phosphorylation [34]. To test whether itraconazole affects FAK dimerization, we overexpressed GST-FAK and examined the interaction between GST-FAK and endogenous FAK. Pulling down GST-FAK co-precipitates endogenous FAK, confirming that they form dimers (Fig. 4D). Itraconazole treatment drastically reduces the amount of co-precipitated endogenous FAK, suggesting that itraconazole disrupts FAK dimerization (Fig. 4D).

5-InsP_7_ strengthens FAK dimer formation, because GST-FAK co-precipitates substantially less endogenous FAK in the *IP6K1* KO cells compared to WTs (Fig. 4E). Pharmacologic inhibition of IP6K1 enzymatic activity by TNP treatment also reduces the binding of GST-FAK with endogenous FAK (Fig. 4F).

5-PCP resin pulls down endogenous FAK in HUVECs (Fig. 4G) and in both WT and *IP6K1* KO MEF cells (Fig. 4H), suggesting that 5-InsP_7_ binds FAK. 5-InsP_7_ has been shown to bind PH domain- and FERM domain-containing proteins [28], [35], and the F3 lobe of the FAK FERM domains exhibit PH domain structure [36]. The *in vitro* protein binding assay demonstrates that 5-InsP_7_ binds FAK at its FERM domain (Fig. 4I).

We performed *in vitro* protein binding assays to confirm that 5-InsP_7_ directly mediates FAK dimerization. 5-InsP_7_, but not its nonhydrolyzable analogs 5-PCP and CF2, enhances the formation of FAK dimer (Fig. 4J-M). Besides, 5-PCP and CF2 compete with 5-InsP_7_ to block 5-InsP_7_-mediated FAK dimer formation (Fig. 4N), indicating that pyrophosphorylation plays a role in 5-InsP_7_-mediated FAK dimer formation.

### 3.5 Itraconazole reduces focal adhesion turnover

**Fig. 5:**
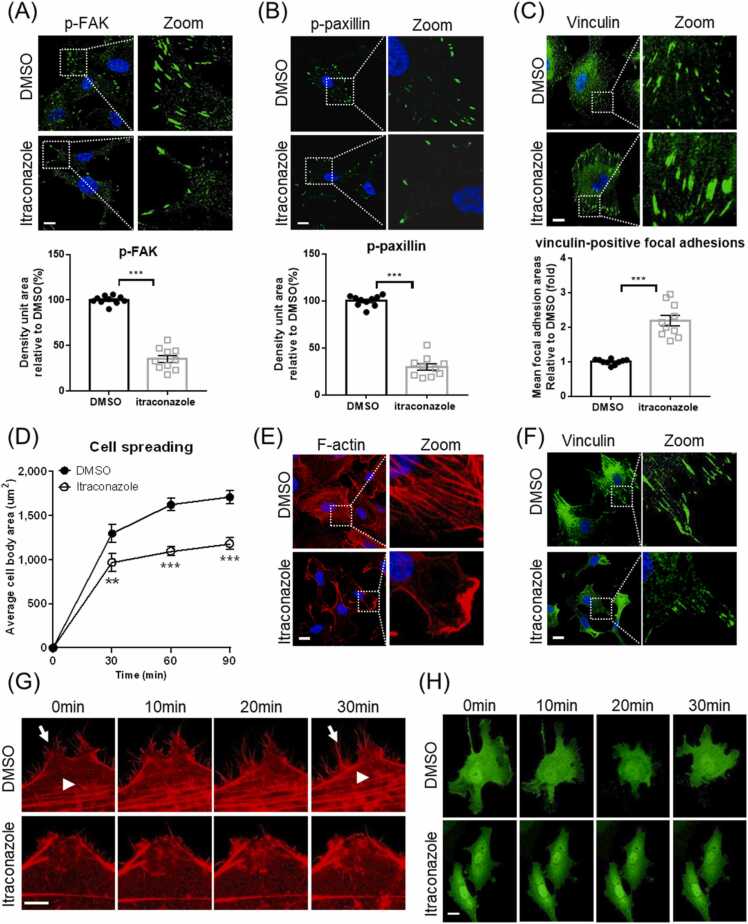
Itraconazole treatment disrupts focal adhesion turnover and arrests cell movement. (A-C) HUVECs were treated with itraconazole (3 μM) for 24 h. (A) Confocal microscopy shows that itraconazole decreases phosphorylated FAK (p-FAK) density. Scale bar 20 µm. (B) Confocal microscopy shows that itraconazole decreases phosphorylated paxillin (p-paxillin) density. Scale bar 20 µm. (C) Immunostaining of vinculin for focal adhesions. The sizes of focal adhesions are considerably larger in the itraconazole treated cells. Scale bar 20 µm. (D-F) HUVECs were treated with itraconazole (3 μM) for 24 h, and were planted onto fibronectin-coated plates. (D) The average body area of the itraconazole-treated spreading cells is decreased. (E) Phalloidin staining demonstrates defective actin stress fiber formation in the itraconazole-treated spreading cells. Scale bar 20 µm. (F) Vinculin staining reveals fewer focal adhesions in the itraconazole-treated spreading cells. Scale bar 20 µm. (G) Live cell imaging of LifeAct-expressing HUVECs. Control cells display F-actin assembly (arrow head) and disassembly (arrow). F-actin remodeling is substantially delayed in itraconazole treated cells. Scale bar 5 µm. (H) Live cell imaging of GFP-expressing HUVECs demonstrates that itraconazole treatment arrests cell movement. Scale bar 20 µm. Statistical data are presented as means ± SEM, Student’s *t*-test, 10 images from 5 independent experiments were analyzed, * *P < 0.01, * **P < 0.001.

Confocal microscopy shows that the density of phosphorylated FAK is markedly decreased in itraconazole-treated HUVECs (Fig. 5A). Similarly, the density of phosphorylated paxillin, a downstream target of FAK, is diminished in itraconazole-treated HUVECs (Fig. 5B). The sizes of focal adhesions, as evidenced by vinculin staining, are relatively larger in the itraconazole-treated cells, indicating fewer turnovers of focal adhesions (Fig. 5C).

Cell spreading requires dynamic reorganization of actin cytoskeleton and coordination of FAK phosphorylation, and is delayed in itraconazole-treated cells (Fig. 5D). At 60 min after plating, phalloidin staining reveals networks of actin filaments in control cells. In contrast, actin filaments exclusively assemble at the cell cortex of itraconazole-treated HUVECs (Fig. 5E), and vinculin staining reveals fewer focal adhesions assemble in itraconazole-treated spreading HUVECs (Fig. 5F).

We double stained phosphorylated FAK and vinculin to validate the role of IP6K1 in itraconazole-induced reduction of focal adhesion turnover (Fig S9). Deletion of IP6K1 reduces FAK phosphorylation, and itraconazole decreases FAK phosphorylation in WT MEF cells but does not further decrease it in *IP6K1* KO MEF cells (Fig S9A). Similarly, deletion of IP6K1 reduces paxillin phosphorylation, and itraconazole lowers phosphorylation levels of paxillin in WT MEF cells but does not further reduce it in *IP6K1* KO MEF cells (Fig S9B).

### 3.6 Itraconazole disrupts actin remodeling and arrests cell movement

**Fig. 6:**
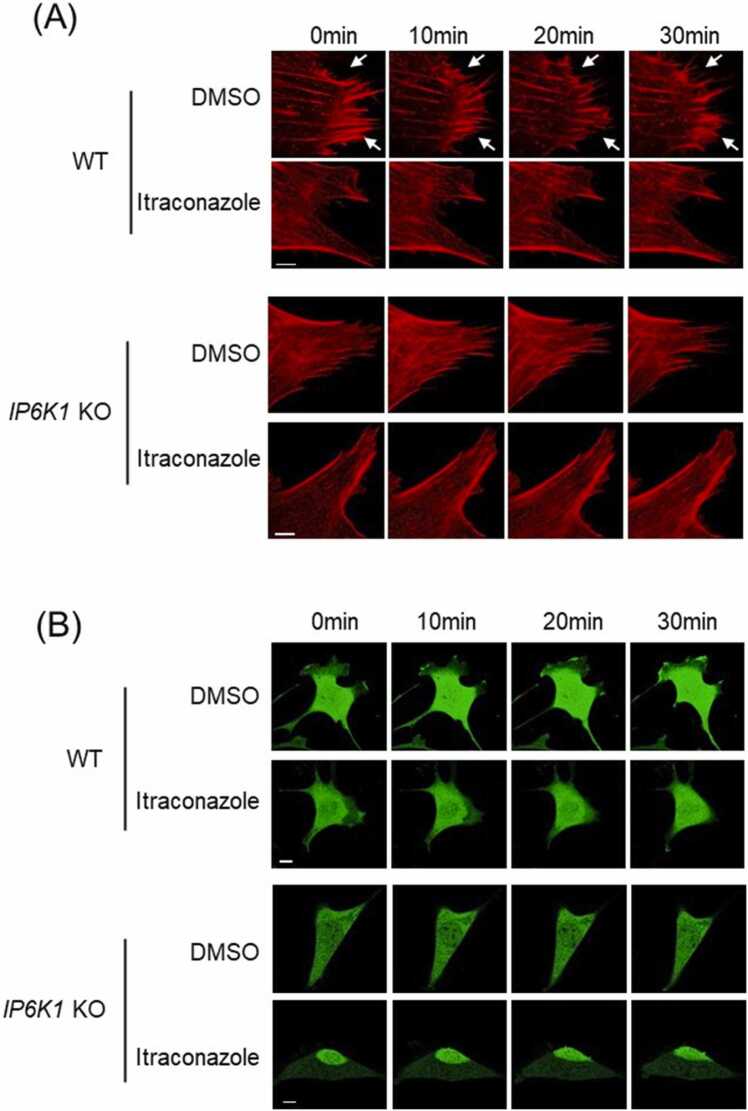
Itraconazole reduces active actin remodeling and delays cell motility in WT but not *IP6K1* KO cells. (A) LifeAct-expressing WT and *IP6K1* KO MEF cells were treated with itraconazole (3μΜ) or DMSO for 24 h. Live cell imaging reveals that active actin remodeling is delayed in *IP6K1* KO cells, and itraconazole delays F-actin remodeling in WT cells but does not further delay it in *IP6K1* KO cells. Arrows point to the assembly and disassembly of F-actin in DMSO treated WT cells. Scale bar 5 µm. (B) GFP over-expressing WT and *IP6K1* KO MEF cells were treated with itraconazole (3μΜ) and DMSO for 24 h. Live cell imaging demonstrates that cell motility is retard in *IP6K1* KO cells, itraconazole delays cell motility in WT cells but not further delay it in *IP6K1* KO cells. Scale bar 20 µm.

We utilized Lifeact-mScarlet to label actin filaments in living cells and monitored actin dynamics under the confocal microscope (Fig. 5G, Video 1, 2). Itraconazole treatment severely impedes the active remodeling of actin filaments. Over a 30-minute period, the control cells displayed assembly and disassembly of actin filaments (Fig. 5G, Video 1). In striking contrast, few changes of actin filaments were observed in the itraconazole-treated HUVECs (Fig. 5G, Video 2). We utilized fluorescence microscopy to monitor the cell movement of GFP-expressing HUVECs. The control cells display lamellipodia protrusion and retraction (Fig. 5H, Video 3), but itraconazole-treated cells adhere tightly to the cell culture plate and barely move (Fig. 5H, Video 4). We also confirmed that IP6K1 is involved in the itraconazole-elicited disruption of cytoskeletal remodeling and cell motility (Fig. 6, Video 5–12). Deletion of IP6K1 disrupts active actin remodeling (Fig. 6A, Video 5–8). Itraconazole reduces active actin remodeling in WT MEF cells but does not further reduce it in *IP6K1* KO MEF cells (Fig. 6A, Video 5–8). Similarly, deletion of IP6K1 impairs cell motility (Fig. 6B, Video 9–12). Itraconazole delays cell movement in WT MEFs but does not further delay it in *IP6K1* KO MEF cells (Fig. 6B, Video 9–12).

Supplementary material related to this article can be found online at doi:10.1016/j.biopha.2023.114449.

The following is the Supplementary material related to this article Video 1, Video 2, Video 3, Video 4, Video 5, Video 6, Video 7, Video 8, Video 9, Video 10, Video 11 and Video 12..

Video 1 lifeact DMSO.

Video 2 life act itraconazole.

Video 3 cell movement DMSO.

Video 4 cell movement itraconazole.

Video 5 WT lifeact DMSO.

Video 6 WT lifeact itraconazole.

Video 7 KO lifeact DMSO.

Video 8 KO lifeact itraconazole.

Video 9 WT cell movement DMSO.

Video 10 WT cell movement itraconazole.

Video 11 KO cell movement DMSO.

Video 12 KO cell movement itraconazole.

## 4 Discussion and conclusions

**Fig. 7:**
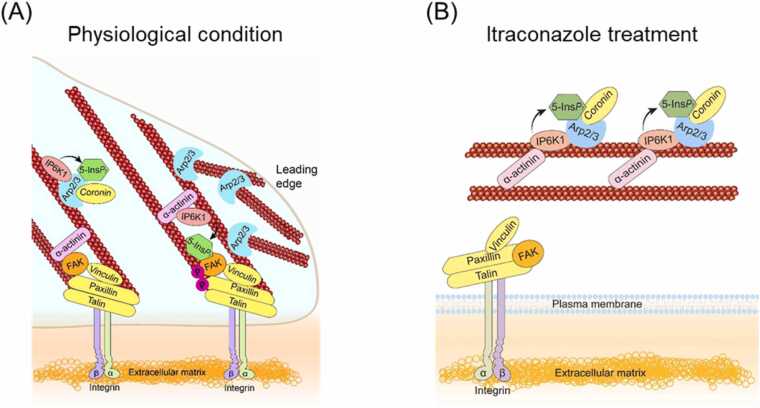
Model for itraconazole disruption of 5-InsP_7_-mediated focal adhesion and actin filaments remodeling. (A) Physiologically, IP6K1 generates a local pool of 5-InsP_7_ in focal adhesions to promote FAK dimerization and phosphorylation. Some IP6K1 protein also binds Arp2 and generates 5-InsP_7_ to mediate the binding of coronin to Arp2/3 complex. (B) Itraconazole treatment redistributes IP6K1 from focal adhesions to Arp2/3 complex, simultaneously reducing 5-InsP_7_-mediated FAK activation and enhancing 5-InsP_7_-mediated Arp2/3 inhibition.

Despite significant efforts to repurpose the anti-fungal drug itraconazole as an anti-angiogenic agent, the mechanisms of action have been elusive. Endothelial cell proliferation and migration are essential components of angiogenesis. In this study, we demonstrate a functional mechanism by which itraconazole inhibits endothelial cell migration. Itraconazole treatment disrupts active remodeling of focal adhesions and the actin cytoskeleton, which requires IP6K1 and its product 5-InsP_7_. IP6K1 generates 5-InsP_7_ at focal adhesions to mediate FAK dimerization and phosphorylation. Some IP6K1 protein also binds Arp2 and generates 5-InsP_7_ to recruit coronin to Arp2/3 complex. Itraconazole treatment shifts IP6K1 from focal adhesion to Arp2/3 complex, simultaneously reducing 5-InsP_7_-mediated FAK phosphorylation and augmenting 5-InsP_7_-mediated recruitment of coronin to Arp2/3 complex (Fig. 7). As a result, itraconazole disrupts 5-InsP_7_-regulated focal adhesion dynamics and actin cytoskeleton remodeling to impede cell motility.

Energetic remodeling of actin filaments and focal adhesions is essential for cell motility. The Arp2/3 complex generates a dendritic actin network at the leading edge of motile cells to form lamellipodia, which are widely believed to be critical for directional migration [26]. Binding of coronin to Arp2/3 promotes disassembly of branched actin networks [37], and it is required for Arp2/3-mediated actin dynamics *in vivo* [38]. The coordination of coronin and Arp2/3 complex plays important roles in the leading-edge actin dynamics and overall cell motility [37]. IP6K1 binds Arp2, and generates 5-InsP_7_ to perform physiological functions. The inhibition of Arp2/3 by coronin is concentration-dependent [38]. Itraconazole-induced redistribution of IP6K1 to Arp2/3 complex strengthens 5-InsP_7_-mediated recruitment of coronin and shift towards Arp2/3 inhibition. This itraconazole-induced Arp2/3 inhibition may negatively feedback the expression levels of coronin, which displays 30% lower in the itraconazole treated cells.

The Arp2/3 complex is regulated by conformational changes [39], [40], [41]. Both ATP and ADP bind Arp2. ATP binding activates Arp2/3 complex [39], [40], whereas ADP binding causes Arp2/3 complex to debranch from mother filaments [42], [43]. Generating 5-InsP_7_ consumes ATP and produces ADP, thus 5-InsP_7_ may function together with ADP to inhibit Arp2/3 complex. We speculate that 5-InsP_7_ may act as a “molecular glue” or cause conformational changes of coronin and the p34 subunit of the Arp2/3 complex to promote the interaction. The details of conformational mechanisms require further structural studies.

The effects of 5-InsP_7_ can be modulated by the activity of diphosphoinositol pentakisphosphate kinase (PPIP5K), which converts 5-InsP_7_ to InsP_8_ [44]. PPIP5K (also named asp1/vip1 in yeast) has long been known to be a critical regulator of Arp2/3 complex, but the mechanism has not been delineated [45], [46]. Because PPIP5K does not physically interact with the Arp2/3 complex, PPIP5K may affect Arp2/3 indirectly by altering 5-InsP_7_ levels [44], [45], [46]. Coherently, deletion of PPIP5K1 decreases cell motility [47].

In living cells, FAK activation is associated with conformational changes [48]. 5-InsP_7_ directly binds FAK via its FERM domain and promotes FAK dimerization, which is critical for FAK phosphorylation and its kinase-dependent functions at focal adhesions [49]. This mechanism partially explains the critical roles of 5-InsP_7_ in focal adhesion turnover [19], [31], [33], [50]. The extremely short half-life of 5-InsP_7_ requires it to be produced near its target proteins. Removing IP6K1 by itraconazole from focal adhesions deprives them of 5-InsP_7_, impeding FAK dimerization. The non-hydrolyzable analogs of 5-InsP_7_ are not able to mediate FAK dimerization, indicating that pyrophosphorylation plays a role. Protein pyrophosphorylation is complex, although it has been discovered for a decade, its physiological functions and mechanisms have been elusive [51], [52], [53], [54], [55], [56]. How pyrophosphorylation affects FAK requires further studies.

Inositol pyrophosphates mediate diverse cellular processes, such as energy production, protein secretion and DNA damage and repair, in particular subcellular areas. Individual IP6K knockout animals display specific phenotypes, and not compensated by other isoforms [57], [58]. The consequences of itraconazole-elicited displacement of 5-InsP_7_ reiterates the criticality of the compartmentalized production of 5-InsP_7_. Relocation of IP6Ks have been reported in several studies. Phosphatidic acid inhibits inositol synthesis by inducing nuclear translocation of IP6K1 [59]. Translocation of IP6K2 from nucleus to cytosol causes cell death [60]. The direct target of itraconazole in mediating IP6K1 redistribution is currently unknown, and the mechanism by which itraconazole induces redistribution of IP6K1 requires further studies. It is worth noting that itraconazole does not affect the protein levels nor the kinase activity of IP6K1.

The effects of IP6K1/5-InsP_7_ in angiogenesis can be complex. Deleting IP6K1 or depleting 5-InsP_7_ increases glycolysis and activates Akt and AMPK pathways [16], [58], [61], [62], which are known to promote angiogenesis. On the other hand, depleting 5-InsP_7_ disrupts focal adhesion turnover [19], [31], [33], [50], which would impair blood vessel formation. The physiological roles of IP6K1/5-InsP_7_ in angiogenesis are currently unknown, and require systemic studies.

Our study demonstrates that IP6K1-generated 5-InsP_7_ is a critical regulator of focal adhesion dynamics and actin cytoskeleton remodeling, and it reveals a functional mechanism by which itraconazole inhibits cell motility.

## Funding

This work was supported by the 10.13039/501100001809National Natural Science Foundation of China (82070259, 81870232 and 82220108021) and sponsored by 10.13039/100007219Natural Science Foundation of Shanghai (22ZR1440700). W.C. was supported by 10.13039/501100001809National Natural Science Foundation of China (81901162) and 10.13039/501100013105Shanghai Rising-Star Program (20QA1406300). Z.G. was supported by Tianjin University of Traditional Chinese Medicine Young Eagle Program (XJS2022107). A.C.C. was supported by the NIH Medical Scientist Training Program Training Grant T32GM007739. B.V.L.P is a Wellcome Trust Senior Investigator. This research was funded in part by the Wellcome Trust. For the purpose of Open Access the authors have applied a CC BY public copyright license to any Author Accepted Manuscript version arising from this submission.

## CRediT authorship contribution statement

**Ji Qi：**Methodology, Investigation, Data curation. **Weiwei Cheng：**Investigation, Writing – original draft preparation, Funding acquisition. **Zhe Gao：**Investigation, Validation, Funding acquisition. **Yuanyuan Chen：**Investigation. **Megan L. Shipton：**Resources. **David Furkert：**Resources. **Alfred C. Chin：**Writing – review & editing. **Andrew M. Riley：**Resources, Writing – review & editing. **Dorothea Fiedler：**Resources, Writing – review & editing. **Barry V. L. Potter：**Resources, Writing – review & editing. **Chenglai Fu：**Conceptualization, Supervision, Project administration, Writing – review & editing, Funding acquisition.

## Conflict of interest

The authors declare that they have no conflict of interest.

## Data Availability

Data will be made available on request.
