# Supplementary material for: Itraconazole inhibits endothelial cell migration by disrupting inositol pyrophosphate-dependent focal adhesion dynamics and cytoskeletal remodeling
Source: Biomed Pharmacother. 2023 May;161:114449. doi: 10.1016/j.biopha.2023.114449 (PMC7614367; doi:10.1016/j.biopha.2023.114449)
Supplement: Supplementary file 13 — Supplementary material [file mmc1.docx]

**Supplemental Figs**

**
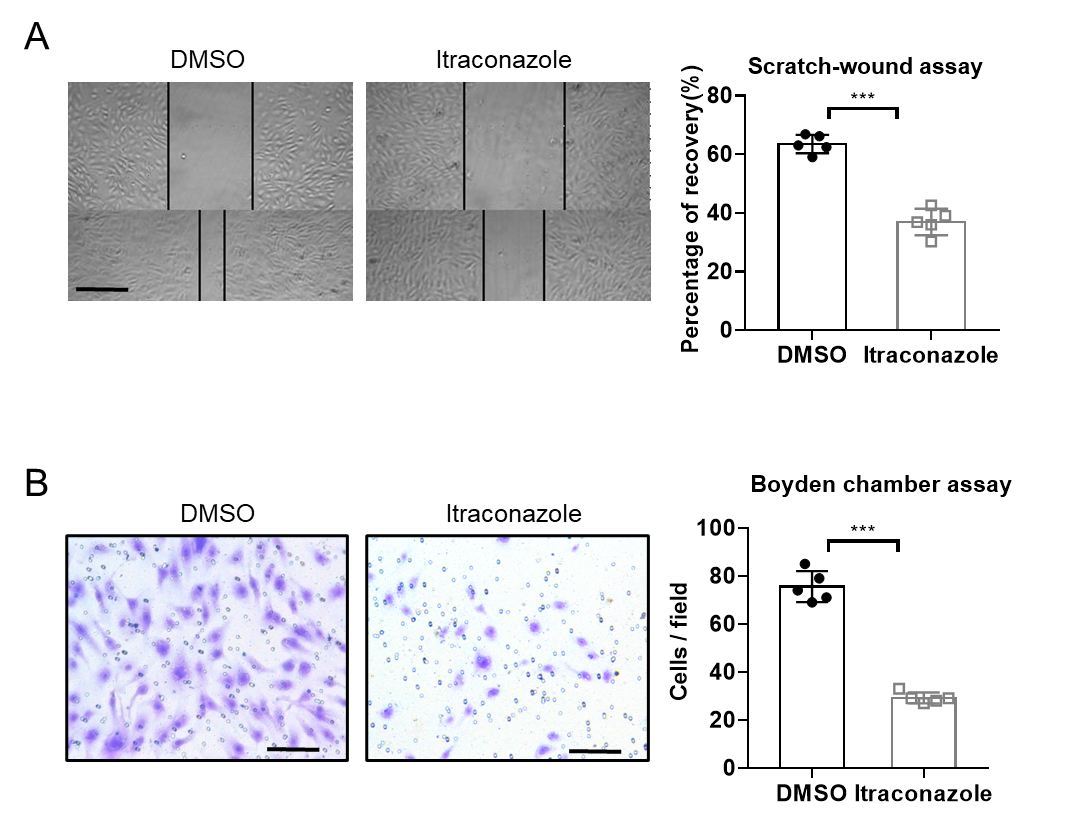
**

**Fig S1. Itraconazole inhibits endothelial migration.**

HUVECs were treated with DMSO or itraconazole (3μΜ) for 24 h. (A) Scratch wound healing assay shows that itraconazole substantially inhibits endothelial migration. Statistical data are presented as mean ±SEM, ***p<0.001, Student’s *t*-test, n=5 independent repeats. Scale bar 200μm. (B) Boyden chamber assay shows that drastically fewer itraconazole-treated cells migrate to the lower chamber. Statistical data are presented as mean ±SEM, Student’s *t*-test, n=5 independent repeats, ***p<0.001. Scale bar 100μm.

**
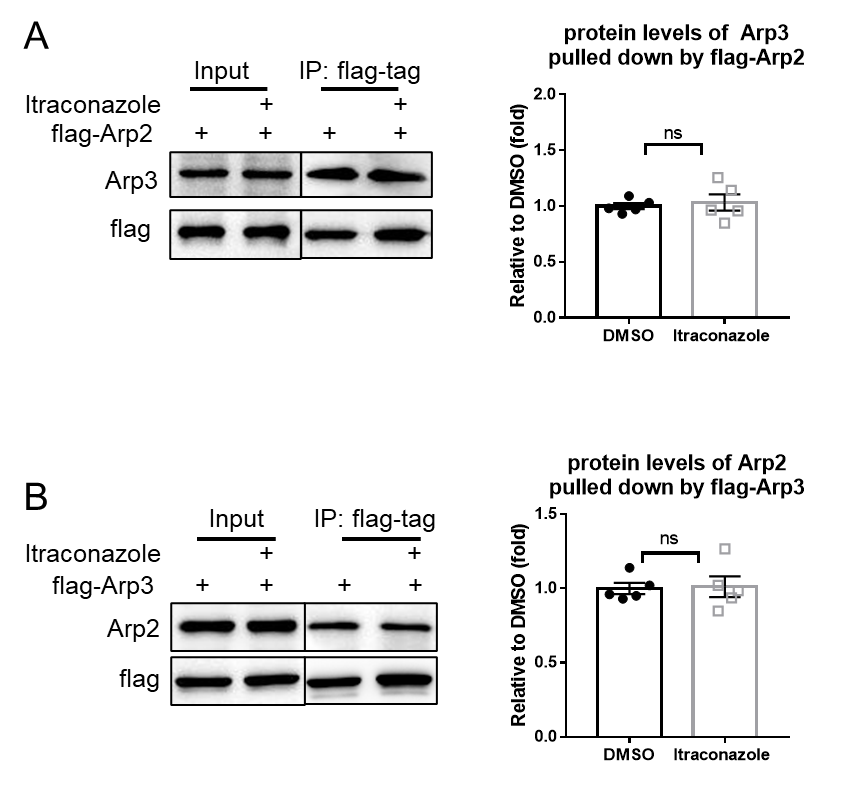
**

**Fig S2. Itraconazole does not affect the interaction between Arp2 and Arp3.**

(A) Flag-Arp2 was overexpressed, and the cells were treated with DMSO or itraconazole (3μΜ) for 24h. Immunoprecipitation of flag-Arp2 co-pulls down similar amounts of Arp3 in DMSO- and itraconazole-treated cells. (B) Flag-Arp3 was overexpressed, and the cells were treated with DMSO or itraconazole (3μΜ) for 24h. Immunoprecipitation of flag-Arp3 co-pulls down similar amounts of Arp2 in DMSO- and itraconazole-treated cells. Statistical data are presented as mean ±SEM, Student’s *t*-test, n=5 independent repeats, ns=not significant.

**
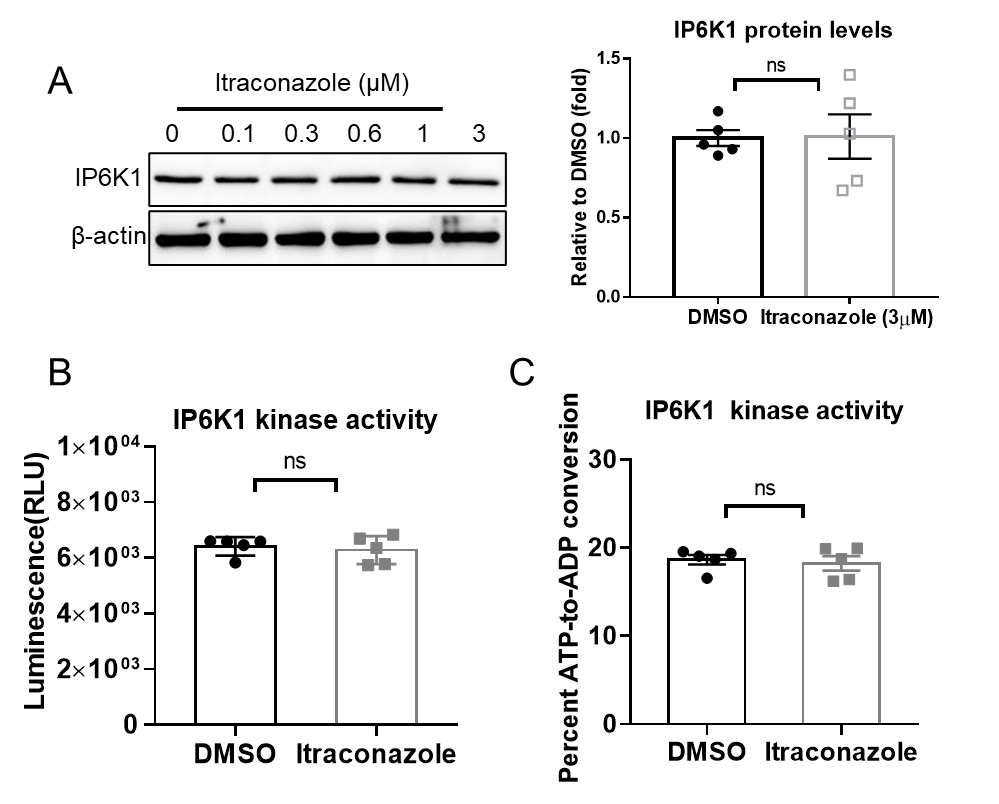
**

**Fig S3. Itraconazole does not affect IP6K1 protein level nor its kinase activity.**

(A) HUVECs were treated with itraconazole for 24h. Itraconazole treatment does not affect IP6K1 protein levels. (B and C) Purified IP6K1 protein was incubated with DMSO or itraconazole in an *in vitro* kinase activity assay. IP6K1 activity was measured via luminescence signal correlating with ADP production. Itraconazole does not affect IP6K1 kinase activity. Statistical data are presented as mean ±SEM, Student’s *t*-test, n=5 independent repeats, ns=not significant.


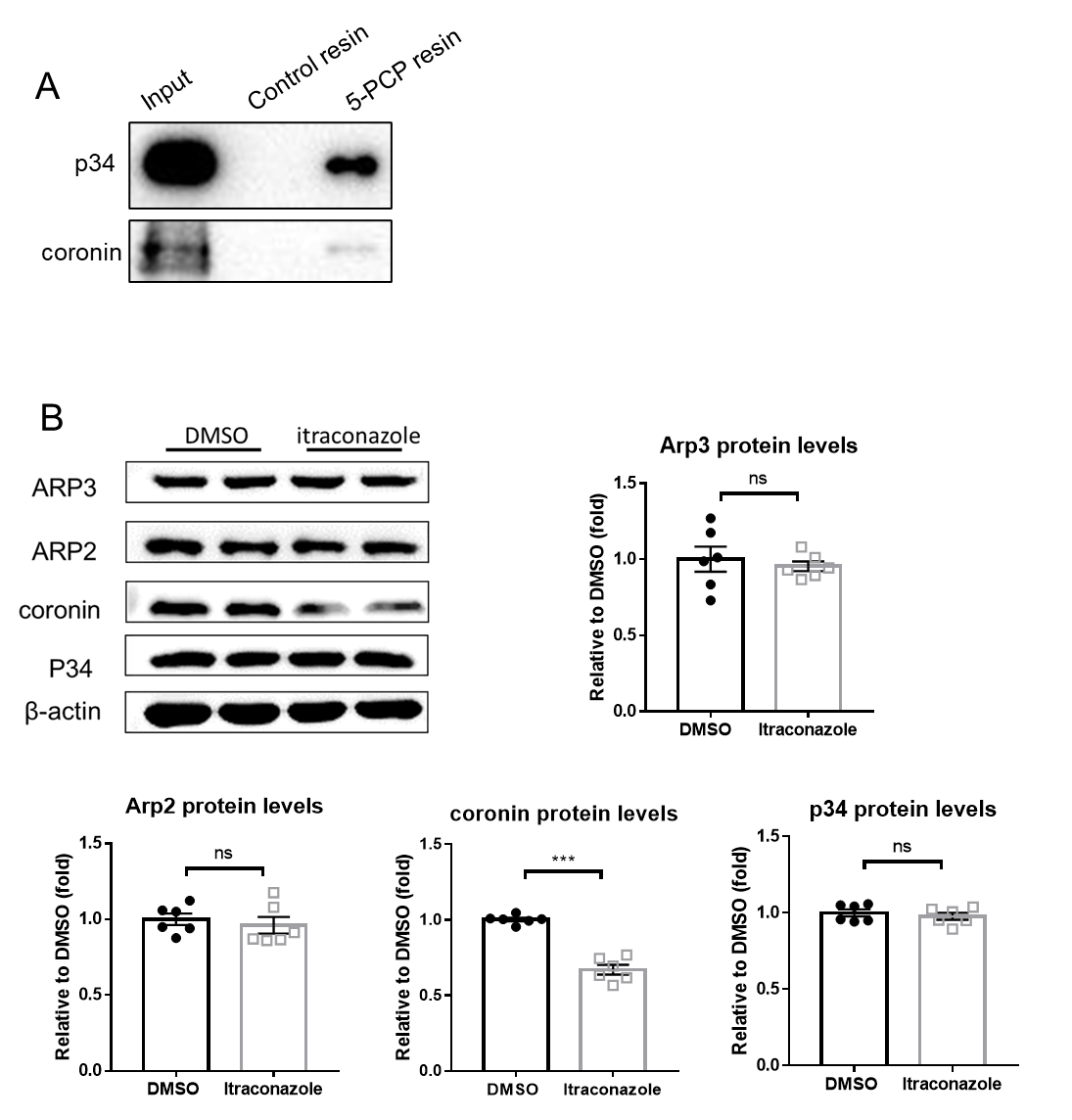


**Fig S4. 5-InsP_7_ binds p34 and coronin.**

(A) 5-PCP resin was incubated with purified p34 or coronin in an *in vitro* protein binding assay. Western blot shows that 5-PCP resin pulls down p34 and coronin, indicating that 5-InsP_7_ binds p34 and coronin. (B) HUVECs were treated with itraconazole for 24h. Western blots show that the expression levels of Arp2, Arp3 and P34 are similar in DMSO and itraconazole treated cells. The expression levels of coronin are 30% lower in the itraconazole treated cells. Statistical data are presented as mean ±SEM, Student’s t-test, n=6 independent repeats, ***P<0.001, ns=not significant.

**
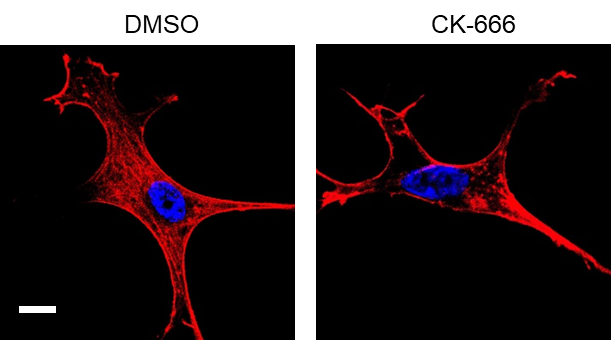
**

**Fig S5. Inhibiting Arp2/3 elicits alteration of actin filament architectures.**

HUVECs were treated with CK-666 (100μΜ) or DMSO for 0.5h. Fluorescein phalloidin staining reveals that F-actin is largely accumulated at the cell cortex in the CK-666-treated cells. Scale bar 20μm.


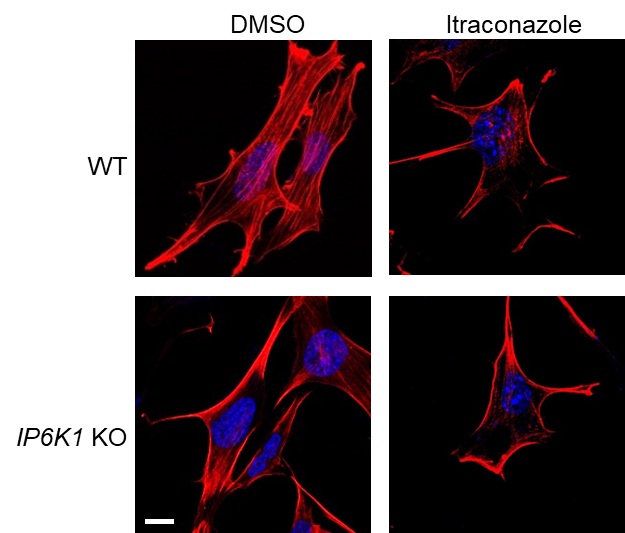


**Fig S6. Itraconazole elicits alteration of actin filament architectures in WT but not *IP6K1* KO cells.**

WT and *IP6K1* KO MEF cells were treated with itraconazole (3μΜ) or DMSO for 24h. Fluorescein phalloidin staining reveals that deletion of IP6K1 impairs F-actin formation, and itraconazole elicits redistribution of F-actin in WT but not *IP6K1* KO cells. Scale bar 20μm.


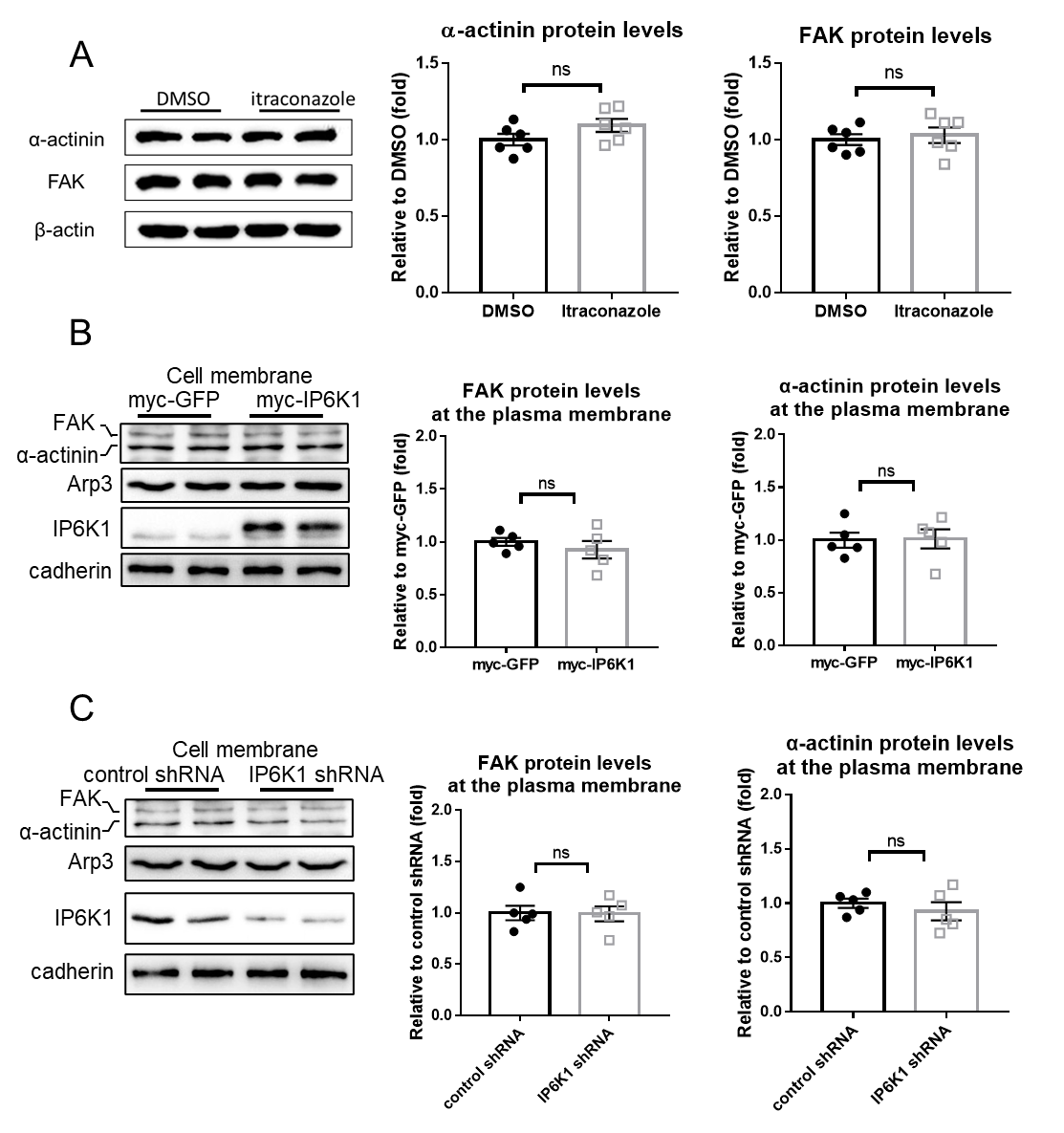


**Fig S7. IP6K1 does not affect the plasma membrane localization of α-actinin.**

(A) HUVECs were treated with itraconazole for 24h. The expression levels of α-actinin and FAK are similar in DMSO and itraconazole treated cells. Statistical data are presented as mean ±SEM, Student’s t-test, n=6 independent repeats, ns=not significant. (B) Overexpressing IP6K1 does not increase α-actinin protein levels in the cell membrane fractions. Statistical data are presented as mean ±SEM, Student’s *t*-test, n=5 independent repeats, ns=not significant. (C) Deletion of IP6K1 does not increase α-actinin protein levels in the cell membrane fractions. Statistical data are presented as mean ±SEM, Student’s *t*-test, n=5 independent repeats, ns=not significant.


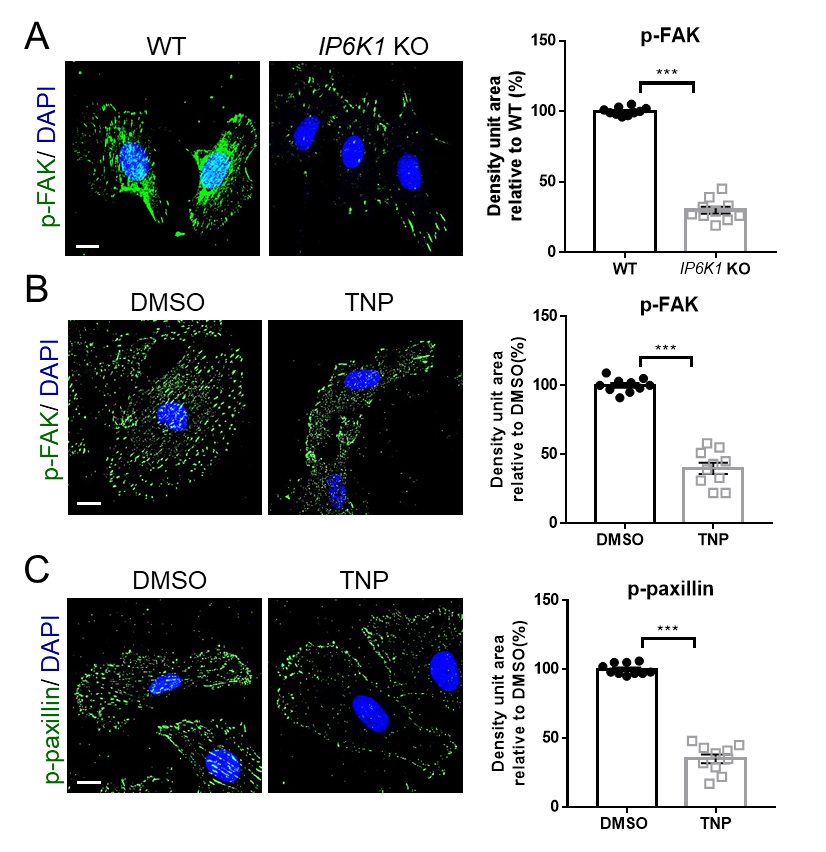


**Fig S8. Inhibiting IP6K1 kinase activity reduces phosphorylation levels of FAK and paxillin.**

(A) Immunostaining of phosphorylated FAK (p-FAK) in WT and *IP6K1* KO MEF cells. The levels of p-FAK are reduced in *IP6K1* KO cells. Scale bar 20μm. (B and C) HUVECs were treated with TNP (5μΜ) or DMSO for 24h. (B) Cells were immunostained with anti-p-FAK. The levels of p-FAK are reduced in itraconazole-treated cells. Scale bar 20μm. (C) Cells were immunostained with anti-p-paxillin antibodies. The levels of p-paxillin are reduced in itraconazole-treated cells. Scale bar 20μm. Statistical data are presented as means ± SEM, Student’s *t*-test, 10 images from 5 independent experiments were analyzed, ***P<0.001.


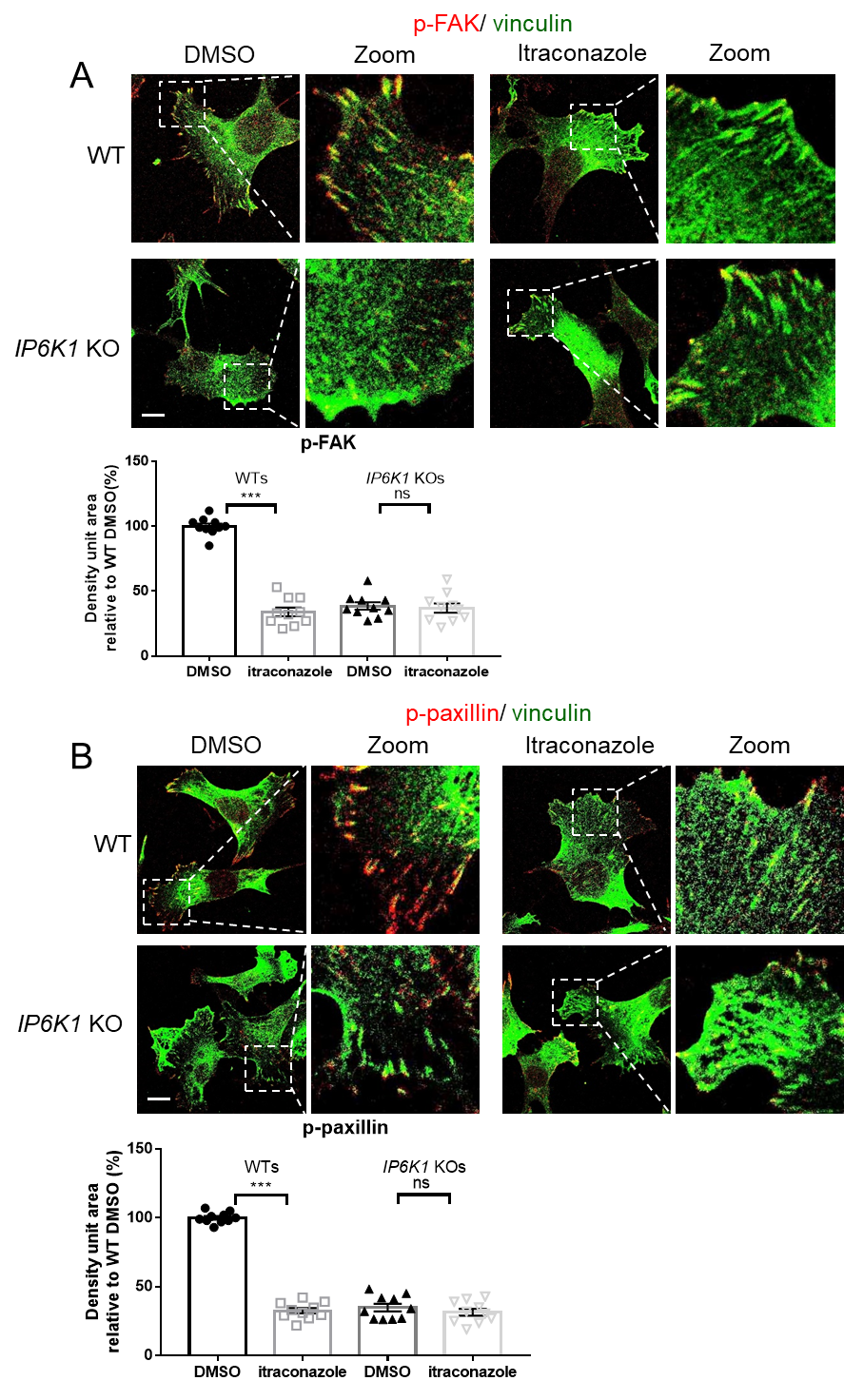


**Fig S9. Itraconazole reduces phosphorylation levels of FAK and paxillin in WT but not *IP6K1* KO cells.**

WT and *IP6K1* KO MEF cells were treated with itraconazole (3μΜ) or DMSO for 24h. (A) Immunostaining of phosphorylated FAK (p-FAK) and vinculin. The levels of p-FAK are less in *IP6K1* KO cells. Itraconazole reduces the levels of p-FAK in WTs but does not further reduce it in *IP6K1* KO cells. Scale bar 20μm. (B) Immunostaining of phosphorylated paxillin (p-paxillin) and vinculin. The levels of p-paxillin are less in *IP6K1* KO cells. Itraconazole reduces the levels of p-paxillin in WTs but does not further reduce it in *IP6K1* KO cells. Scale bar 20μm. Statistical data are presented as means ± SEM, Student’s *t*-test, 10 images from 5 independent experiments were analyzed, ***P<0.001, ns=not significant.
